# Yi Shen An, a Chinese traditional prescription, ameliorates membranous glomerulonephritis induced by cationic bovine serum albumin in rats

**DOI:** 10.1080/13880209.2021.2021947

**Published:** 2022-01-08

**Authors:** Yun-Li Zhao, Xiang-Hua Zhang, Feng Guo, Ying Wei, Jian-Hua Shang, Xiao-Dong Luo

**Affiliations:** a Key Laboratory of Medicinal Chemistry for Natural Resource, Ministry of Education, Yunnan Provincial Center for Research & Development of Natural Products, School of Chemical Science and Technology, Yunnan University, Kunming, P. R. China; b New Drug R&D Department of Kunming Institute of Kidney Disease, Kunming, P. R. China; c Shang Hai University of Medicine & Health Sciences, Shang Hai, P. R. China; d State Key Laboratory of Phytochemistry and Plant Resources in West China, Kunming Institute of Botany, Chinese Academy of Sciences, Kunming, P. R. China

**Keywords:** Nephritis, circulating immune complex, proteinuria, podocytes

## Abstract

**Context:**

Yi Shen An (YSA) is an investigational composite of traditional Chinese medicine (Reference: 2010L000974) for the treatment of renal disease.

**Objective:**

To investigate the protective effects of YSA against membranous glomerulonephritis (MGN).

**Materials and methods:**

Male Sprague–Dawley rats were injected with cationic bovine serum albumin (C-BSA) to create a model of MGN. Then, rats were orally treated with YSA at doses of 0.25, 0.5, 1 and 2 g/kg for 35 successive days; prednisone (5 mg/kg) was used as a positive control. At the end of the experimental period, we performed a series of tests, including 24 h urinary protein, and biochemical, immunological, antioxidative, coagulation indices, and histopathological examination.

**Results:**

YSA-1 g/kg significantly lowered urinary protein from 68.37 to 30.74 mg (*p* < 0.01). Meantime, total protein (TP) and albumin (ALB) recovered from 66.26 and 20.51 g/L to 76.08 and 35.64 g/L (*p* < 0.01), respectively. YSA removed the deposition of immunoglobulin G (IgG) and complement 3c (C3c), prevented inter-capillary cell hyperplasia on the glomerular basement membrane (GBM), and reduced electron-dense deposits and fusion of podocytes. In addition, serum IgG and superoxide dismutase were significantly elevated. In contrast, malondialdehyde, total cholesterol, triglyceride, circulating immune complex (CIC), and immunoglobulin M decreased in the YSA-treated group. Moreover, the blood coagulation dysfunction was adjusted.

**Discussion and conclusions:**

These findings indicate YSA may exert a therapeutic effect against MGN through the inhibition of CIC formation, and the removal of IgG and C3c deposition from the GBM, thus supporting the development of further clinical trials.

## Introduction

Glomerulonephritis is a major contributing factor to end-stage renal damage. Approximately 30–40% of patients develop progressive renal impairment, thus resulting in end-stage renal failure after 10–15 years (Glassock 2003; Remuzzi et al. 2006). Membranous nephropathy, widely known as membranous glomerulonephritis (MGN), is the most common cause of glomerulonephritis in adults, with a reported annual incidence ranging from 0.2% to 1.4%, and an overall estimate of 1.2% annually (Horvatić and Galesić 2012; Kronbichler et al. 2017). MGM is defined by the deposition of immune complexes on the epithelial side of the glomerular capillary wall (Ronco and Debiec 2011), glomerular basement membrane (GBM), and podocytes, thus causing chronic primary glomerular disease with diffuse thickening of the GBM (Border et al. 1982; Song et al. 2016).

Exogenous or endogenous antigens may stimulate the body to produce antibodies, thus forming circulating immune complexes (CICs) in the blood (Adler et al. 1983). Subsequently, these CICs are deposited in the GBM, thereby activating the complement system and inflammatory response. This process eventually leads to the development of glomerulonephritis (Emancipator et al. 1985). The CICs formed by antigens and antibodies are deposited in the glomeruli. Furthermore, the specific site of deposition is known to be dependent on the size and charge of the CICs, as well as the type and affinity of the antibody (Wang et al. 2006). The formation of immune complexes represents the first step in the pathogenesis of MGN (Sasaki et al. 1997). Glomerular damage and clinical symptoms occur in the presence of single or multiple inflammatory mediators (Sato et al. 1999; Kriz et al. 2003). These mediators include activated complement and its cleaved fragments. The main sources of oxygen-free radicals in the kidney tissues are intrinsic and infiltrating kidney cells (Fulpius et al. 1996; Hebert et al. 1998). The generation and elimination of oxygen-free radicals in local renal tissue is an important mechanism in the development of chronic kidney disease (Liu et al. 2012). Increased levels of oxygen-free radical activity may cause damage to the structure and function of the cell membrane, aggravate glomerulonephritis, and eventually lead to glomerular sclerosis, renal tubular atrophy, and interstitial fibrosis. The availability of oxygen-free radical scavengers, or antioxidant enzymes, may relieve this form of kidney disease (Moon et al. 2012). Coagulation or the formation of microthrombi in the glomerular capillaries is a common feature in the pathogenesis of nephropathy. Hyperlipidaemia, hyper-viscosity, and hypercoagulability, are commonly detected by hemorheology (Llach 1985; Kayali et al. 2008). In addition, the degree of hypercoagulability is known to be dependent on the severity and activity of nephropathy (Llach 1985). The pathogenic factors associated with nephritis, as described above, are also involved in the development of nephritis and can exert effect throughout the entire course of nephritis.

Despite the progress achieved relating to the pathogenesis, clinical manifestations, diagnosis, and treatment of MGN, there is no satisfactory treatment for this disease at present. Clinically, the currently available treatment options for MGN include hormone therapy, immunosuppressive agents, cytotoxin, anticoagulants, and angiotensin-converting enzyme inhibitors/angiotensin II receptor blockers (Kshirsagar et al. 2003; Lai et al. 2015). Despite improvements in these treatments, unfavourable events are commonly reported (Lai et al. 2015; Song et al. 2016), including adverse hematological or metabolic events and infection (Howman et al. 2013), along with liver damage and myelosuppression (Waldman and Austin 2012). Thus, there is an urgent need to develop a safe, effective, and multi-target agent that is capable of curing both the symptoms and causes of MGN.

Traditional Chinese medicine (TCM) is used for the treatment of incurable diseases worldwide. Yi Shen An (YSA), as a treatment against nephritic syndrome, was provided by the Kunming Institute of Kidney Disease, Yunnan, China, and includes *Panax notoginseng* (Burkill) F.H. Chen (Araliaceae), *Rheum officinale* Baill. (Polygonaceae), *Salvia miltiorrhiza* Bunge (Lamiaceae), *Lonicera confusa* (Sweet) DC. (Caprifoliaceae), *Carthamus tinctorius* L. (Compositae), *Forsythia suspensa* (Thunb.) Vahl (Oleaceae), *Scutellaria barbata* D. Don (Labiatae), *Wolfiporia extensa* (Peck) Ginns (Polyporaceae) and *Glycyrrhiza uralensis* Fisch (Leguminosae). Our previous studies showed that YSA could enhance the immune function of immunocompromised mice, reduce blood viscosity, and inhibit platelet aggregation, in a rat model of blood stasis, and improve microcirculation in a mouse model of epinephrine-induced microcirculation disorder. This preparation has been registered as an investigative new botanical drug (Reference: 2010L000974) and approved for phase I/II clinical trials by the China Food and Drug Administration (CFDA). YSA has been tested in many different ways (phase II trials; safety and efficacy trials; and randomized, double-blinded, single simulation, positive and placebo-controlled, multicenter studies; the results of these trials supported further phase-Ш clinical trials.

Therefore, the objective of this study was to further investigate the mechanisms underlying the actions of YSA and its protective effects against MGN induced by cationic bovine serum albumin (C-BSA). Our approach involved the construction of a rat model of immune complex membranous nephritis *via* tail vein injection. Our findings may provide novel insights into the pharmacological actions of YSA as a potential natural drug for the treatment of kidney disease.

## Materials and methods

### Experimental materials

Ten herbs of YSA were purchased from Hongxiang Pharmaceutical Group Co., Ltd. (Yunnan, China) and formally identified by Professor Yong-Ding Sun, Yunnan Medicinal Materials Co., Ltd. (Yunnan, China). Figure S1 (Supporting Information) shows the methods used to extract YSA. The main chemical components of YSA were then identified by high-performance liquid chromatography quadrupole-time-of-flight mass spectrometry ultraviolet (HPLC-Q/TOF MS-UV).

**Figure 1.**
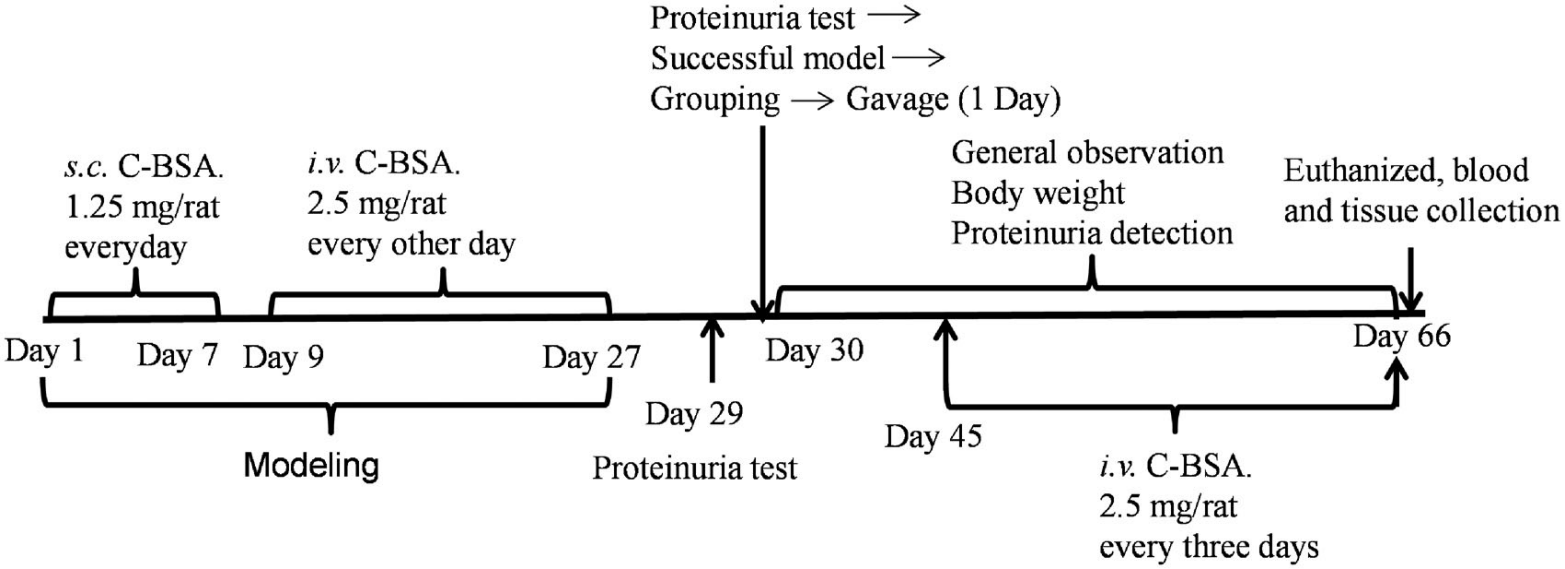
Experimental procedure. *s.c.*, subcutaneous injection; *i.v.*, intravenous injection; C-BSA, cationic bovine serum albumin.

### Reagents

The levels of total cholesterol (TC), triglycerides (TG), urea nitrogen (BUN), serum creatinine (CRE), total protein (TP), and serum albumin (ALB), were detected using specific assay kits from Kehua Bio-engineering Co., Ltd. (Shanghai, China). Malondialdehyde (MDA) and superoxide dismutase (SOD) assay kits were purchased from Nanjing Jiancheng Bioengineering Institute (Nanjing, China). Bovine serum albumin (BSA; 96–99% purity) was purchased from Boao Biotechnology Co., Ltd. (Shanghai, China). Detection kits for immunoglobulins (IgA, IgM, and IgG) were purchased from EURO-DIAGNOSTICA (Amsterdam, Netherlands). Reagent kits for plasma prothrombin time (PT), activated partial thromboplastin time (APTT), thrombin time (TT), and plasma fibrinogen (FIB), were obtained from SUNBIO Technology Co., Ltd. (Shanghai, China). All other chemicals were of reagent grade.

### Animals

Specific-pathogen-free (SPF) male Sprague-Dawley rats, weighing between 180 and 200 g, were purchased from the Laboratory Animal Centre of Kunming Medical University (license number: SCXK K2015-0002). All animals were housed at room temperature (20–25 °C) and constant humidity (40–70%) under a 12 h light-dark cycle in an SPF grade laboratory. Food and water were supplied *ad libitum*. The animals were acclimatized for 1 week prior to experimentation. All animal experiments complied with institutional and governmental regulations regarding the use of experimental animals. The experimental protocol was reviewed and approved by the Institutional Animal Care and Use Committee of the Yunnan Institute of Medical Material (Animal ethics approval code number: YIMM2008-003).

### Preparation of C-BSA

C-BSA was prepared as described previously but with slight modifications (Border et al. 1982; Li et al. 2007). First, an anhydrous ethylenediamine (EDA) solution was produced by mixing 350 mL of EDA and 2.5 L of distilled water. Subsequently, 1.75 L of 6 mol/L HCl was added to adjust the pH to 4.75 prior to cooling the solution to 25 °C with an ice bath. BSA (30 g) was dissolved in 150 mL of distilled water and added to the EDA solution, followed by 12 g of 1-(3-dimethylaminopropyl)-3-ethylcarbodiimide hydrochloride. The reaction was allowed to continue for 6 h with continuous stirring at a constant temperature (25 °C) and a pH of 4.75. The reaction was terminated by the addition of 150 mL of 4 mol/L acetate buffer (pH 4.75).

Subsequently, (NH_4_)_2_SO_4_ was added to the reaction mixture to achieve a saturation index of 0.8. The mixture was then centrifuged for 10 min at 12,000 × *g* to collect the precipitate; this procedure was then repeated with the remaining supernatant. Next, (NH_4_)_2_SO_4_ was added to the supernatant to achieve a saturation index of 0.9. Finally, the entire precipitate was diluted with water to a volume of 150 mL and filtered. The filtrate was then desalted for 72 h by water dialysis at 4 °C (liquid infiltration; pH 6.5–8.0). The filtered concentrate was then freeze-dried to 25 g of C-BSA powder. Finally, we used ultraviolet detection to determine the purity (86.39%) and net yield (72.99%) of the concentrate. We also determined the isoelectric point (9.0) by an isoelectric focussing method.

### Generation of the animal model

According to previously described methods (Salant et al. 1980; Li et al. 2007), we preimmunized rats (1 mL/rat) by multiple-point subcutaneous injections of white emulsion every day for 7 days. The emulsion was obtained by mixing 2.5 mg/mL of sterile C-BSA solution with an equivalent amount of Freund’s incomplete adjuvant. Subsequently, animals were intravenously injected with 1 mL of C-BSA (2.5 mg/mL) every other day until they had received 10 consecutive injections. The 12 rats in the control group received an equal volume of phosphate-buffered saline (PBS). Subsequently, we collected 24 h urinary samples using metabolic cages (Suzhou Fengshi Laboratory Animal Equipment Co. Ltd., Jiangsu, China) to determine urinary protein content. Eighty-eight rats, in which the 24 h urinary protein content was >20 mg, were then assigned to six groups, including a model group (*n* = 15), a positive control group receiving prednisone acetate (5 mg/kg, *n* = 14) (PRE), and four YSA groups (0.25, 0.5, 1, and 2 g/kg; *n* = 14–15). The gavage volume was 10 mL/kg and the test substances were suspended in distilled water. The control and model groups were then treated orally with an equal volume of distilled water for 35 days. The other groups were treated with YSA at appropriate doses for 35 days. On the 15th day, all rats (except those in the control group) received intravenous injections of 1 mL of C-BSA (2.5 mg/mL) once every 3 days until the end of the experiment to maintain high levels of urinary protein in the model. Further details relating to this procedure are shown in Figure 1.

### General status and the detection of urinary protein

During the experimental period, the animals in each group were monitored daily for dietary intake, water consumption, activity, and mental state. In addition, 24 h urinary samples were collected prior to treatment, and after 14, 28 and 35 days of treatment, to monitor variations in protein excretion. Urine samples were centrifuged at 800 × *g* for 15 min, and the supernatant was used to determine protein concentration using the Coomassie Brilliant Blue method.

### Blood sampling

At the end of the study period, all rats were anaesthetized by intraperitoneal injections of ketamine-xylazine solution. Blood samples were then collected from the abdominal aorta. First, a 5 mL sample was taken from each rat to isolate serum. Next, we obtained a 1.3 mL blood sample and added 0.15 mL of sodium citrate anticoagulant solution. An additional 1.35 mL of blood was taken and mixed with 0.15 mL of ethylene diamine tetraacetic acid (EDTA). Serum and plasma samples were subsequently obtained by centrifugation at 4000 × *g* for 15 min at 4 °C. These samples were stored at −80 °C to await analysis (Lemos et al. 2014).

### Determination of biochemical indices

Blood testing, which included the determination of the ALB, TP, TC, TG, CRE, and BUN levels was performed using an automatic biochemical analyzer (Beckman Coulter Co., Ltd., IN, USA). The levels of IgG, IgA, IgM, and CIC were detected by immunofluorescence. The levels of PT, APTT, TT, and FIB were measured using a C2000-4 high-performance blood coagulation analyzer (Beijing Precil Instrument Co., Ltd., Beijing, China). Finally, the levels of SOD and MDA were determined by test kits in accordance with the manufacturer’s instructions.

### Kidney histopathology

Following the collection of blood, the kidneys were dissected, rinsed with cold isotonic saline, and weighed. An index of renal hypertrophy was estimated by comparing the wet weight of the left kidney to the body weight. The tissues were then fixed in 10% neutral formalin and embedded in paraffin for histological evaluation (Ichiki et al. 2005; Bei et al. 2013). All embedded sections were stained using haematoxylin and eosin (H&E), periodic acid-Schiff (PAS), and Masson’s trichrome, and then examined by light microscopy (Ma et al. 2015). A semi-quantitative score (on a scale from 0 to 4+) was then used to evaluate the degree of glomerular vacuolated change or sclerosis. This grading scheme represented the proportion (%) of pathological involvement in the glomeruli, as follows: + (≤25%), ++ (25–50%), +++ (50–75%), and ++++ (75–100%). The pathological scores of kidney damage, evaluated according to the aforementioned criteria, were recorded as 0, 1, 2, 3 and 4 points. The sum of these scores in 100 glomeruli was then expressed as the extent of glomerular injury (Lee and Spargo 1985; Masakazu et al. 1993). The severity of tubular atrophy was designated as normal (−), mild (1+), moderate (2+), or extensive (3+) (Austin et al. 1984). Atrophic changes were identified by assessing the thickening of the tubular basement membranes, with or without degeneration of the tubular epithelial cells. The deposition of periglomerular and peritubular fibrous tissue was determined primarily by Masson’s staining. We also measured the diameters of 10 large glomeruli with dense plaques or small arteries (Bhathena et al. 1985).

### Electron microscopy

Samples of kidney tissue for electron microscopy were fixed using 2.5% glutaraldehyde, 0.22 mmol/L sucrose, and 1% osmium tetroxide. The tissues were then dehydrated using ethanol and embedded in epoxy resin (Guo et al. 2014). Subsequently, sections (1 mm^3^) were double-stained using uranyl and lead citrate. Finally, the ultrastructure of the immune complex deposits was evaluated using a Hitachi H-600 transmission electron microscope (Hitachi, Ltd., Tokyo, Japan).

### Immunohistochemistry

For immunohistochemical staining, the kidney tissues were fixed using 10% neutral buffered formalin, embedded in paraffin, cut into 4 μm sections, and placed onto glass slides. Next, paraffin was removed with xylene and the sections were dehydrated with a graded series of alcohols, followed by three washes with PBS. Next, the sections were incubated with 3.0% peroxide for 10 min to block the activity of endogenous peroxidase. For antigen retrieval, the kidney sections were immersed in a 10 mmol/L solution of citrate buffer and heated to 90 °C for 20 min. After cooling for 20 min, the sections were incubated in blocking solution (5% BSA) for 20 min at room temperature. After incubation at 37 °C for 2 h, the sections were stained using a biotinylated rabbit anti-rat IgG to rat C3c (Wuhan BOSTER Biological Engineering Co., Ltd., Wuhan, China). After washing with PBS, the slides were incubated with labelled streptavidin-biotin reagent. Immunoreactive products were then visualised using a liquid diaminobenzidine substrate chromogen system. Sections were then counterstained with haematoxylin for 15 s; positive areas showed a granular appearance with linear brown/yellow deposits. The most intense positive area in the cortical zone of each section was measured using an Image-Pro^®^ Plus 6.0 colour pathological image analysis system (Media Cybernetics Co., Ltd., Bethesda USA) at a magnification of ×200 to calculate the integral optical density and mean optical density (Liu et al. 2009; Oktem et al. 2012).

### Statistical analysis

Data are presented as mean ± standard error of the mean. Comparisons between two groups were performed using two-tailed Mann–Whitney tests. Multiple comparisons were performed using one-way analysis of variance (ANOVA) with least significant differences. Kruskal–Wallis analysis and Dunn’s *post hoc* test were used for non-parametric analyses. The Chi-squared test, and reference identical unit scores, were used to enumerate the data. All statistical analyses were performed using GraphPad Prism (GraphPad, San Diego, CA, USA) software; *p* < 0.05 denoted statistical significance.

## Results

### HPLC profiling and the main constituents/concentrations of YSA capsules

**Figure 2.**
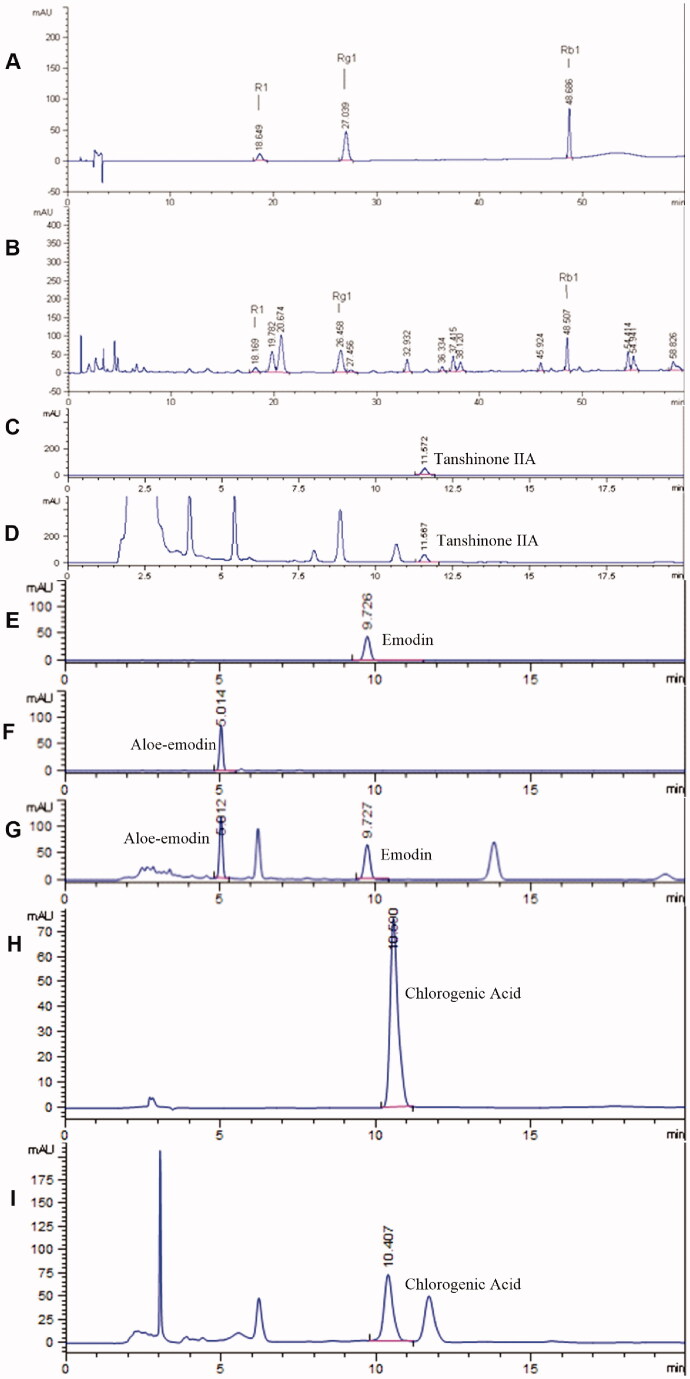
HPLC chromatograms of quality control components. R1, *Panax notoginseng* saponins R1; Rg1, ginseng saponin Rg1; Rb1, ginsenoside Rb1. (A) Reference substance for R1, Rg1, Rb1; (B) R1, Rg1, Rb1 in Yi-shen-An (YSA); (C) Reference substance for tanshinone IIA; (D) Tanshinone IIA in YSA; (E) Reference substance for emodin; (F) Reference substance for aloe-emodin; (G) Emodin and aloe-emodin in YSA; (H) Reference substance for chlorogenic acid; I, Chlorogenic acid in YSA.

Seven chemical components (Figure 2) were identified as the major medicinal agents and quality standards in YSA, including notoginsenoside R1 (retention time [RT] = 18.17 min), ginsenoside Rg1 (RT = 26.46 min), ginsenoside Rb1 (RT = 48.51 min), tanshinone IIA (RT = 8.82 min), emodin (RT = 9.74 min), aloe-emodin (RT = 5.01 min) and chlorogenic acid (RT = 9.59 min). The contents of these constituents were 2.75, 11.53, 8.17, 0.18, 2.09, 1.93, and 4.87 mg/g, respectively.

### General observations

During the study period, some of the animals that had been injected with C-BSA appeared listless and exhibited low activity and poor food intake. These animals were seen to exhibit piloerection, mild diarrhoea, difficulty in breathing; unexpected death was observed occasionally. Four rats died in the model group, three rats in the PRE group, and two, two, three, and three rats in the YSA 0.25, 0.5, 1, and 2 g/kg groups, respectively. Of these, two of the deaths in the YSA-treated group (1 and 2 g/kg) occurred due to inappropriate operator actions. The overall mortality rate induced by C-BSA was 11.4%. Post-mortem anatomical observation revealed the presence of pleural effusion, pulmonary congestion, and abscesses. These observations may have been attributed to the reduced immune function and increased vulnerability to infection after modelling.

In addition, we analyzed the bodyweight of rats at different time points (Table 1). We found that the body weights in all model groups were slightly higher than those observed in the control group prior to the administration of drugs (*p* > 0.05). After treatment, the body weights of animals in the PRE group (5 mg/kg) increased slowly while those of animals in the YSA groups (0.25, 0.5, 1 and 2 g/kg) increased quickly; however, these differences in body weight were not statistically significant (*p* > 0.05). Results derived from kidney index analysis are shown in Table 1. The kidney index was significantly higher in the model group than the control group (*p* < 0.01). As expected, the kidney indices in the YSA groups were significantly lower (*p* < 0.05/0.01) than those observed in the model group.

**Table 1.** The effects of YSA on body weight and kidney index.

Group	0 d (g)	7 d (g)	14 d (g)	21 d (g)	28 d (g)	35 d (g)	Kidney index (g/100 g)
Control	257.43 ± 5.27	264.06 ± 5.90	274.86 ± 5.58	269.47 ± 7.58	289.71 ± 7.88	281.86 ± 6.79	0.6075 ± 0.0173
Model	278.51 ± 10.40	272.31 ± 11.75	302.98 ± 11.41^#^	316.08 ± 11.65^##^	338.82 ± 12.09^##^	322.11 ± 17.99	0.7809 ± 0.0500^##^
PRE	274.33 ± 11.36	264.75 ± 11.62	275.48 ± 11.23	279.10 ± 13.92	301.16 ± 12.98*	302.17 ± 11.39	0.6370 ± 0.0112*
YSA-0.25	280.46 ± 17.44	272.38 ± 19.15	302.30 ± 17.11	310.59 ± 15.60	333.98 ± 14.66	336.95 ± 14.64	0.6273 ± 0.0108*
YSA-0.5	270.91 ± 10.03	266.79 ± 10.09	294.38 ± 9.81	300.37 ± 8.52	324.16 ± 8.46	324.10 ± 7.35	0.6024 ± 0.0152**
YSA-1	275.48 ± 8.34	277.24 ± 8.66	303.25 ± 6.91	308.13 ± 8.96	335.96 ± 7.63	339.49 ± 8.35	0.6363 ± 0.0123*
YSA-2	278.26 ± 9.64	274.91 ± 11.01	295.05 ± 11.92	298.00 ± 10.91	322.79 ± 11.07	328.32 ± 9.55	0.6634 ± 0.0317

Data are shown as means ± standard error of the mean. Statistical differences are represented as ^#/##^*p* < 0.05/0.01 vs. Control, ^*/**^p < 0.05/0.01 vs. Model group. YSA, Yi Shen An. PRE, prednisone acetate positive control. YSA-0.25, 0.5, 1, and 2 represents rats that were orally administrated with YSA at doses of 0.25, 0.5, 1, and 2 g/kg, respectively.

### Quantification of 24 h urinary protein levels

**Figure 3.**
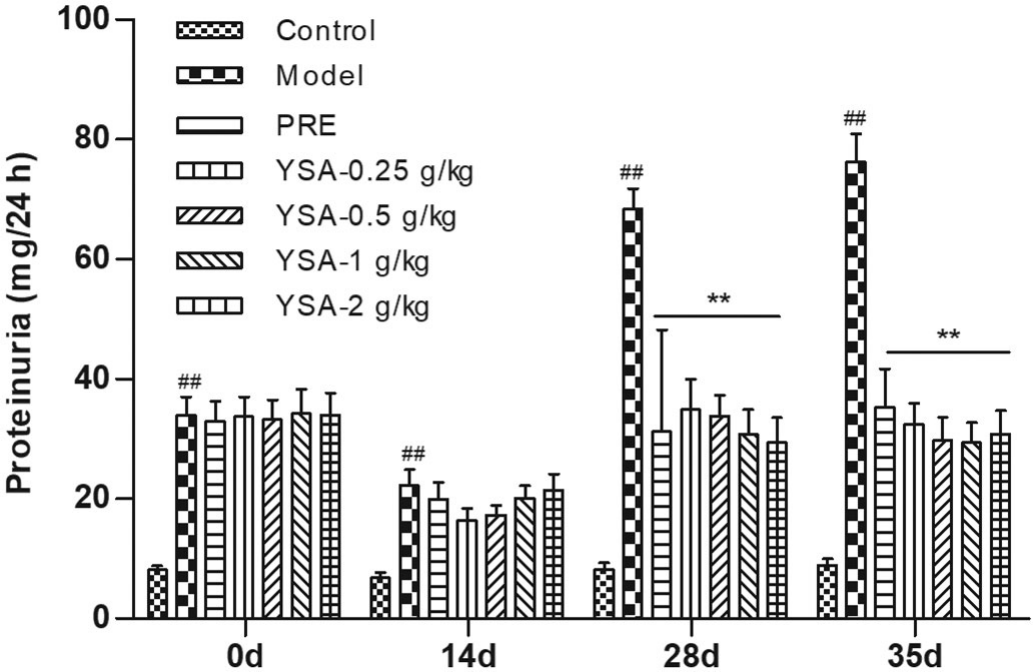
Bar graphs representing levels of 24 h urinary protein. Data are shown as means ± standard error of the mean. Statistical differences are represented as ^##^*p* < 0.01 vs. Control, **p < 0.01 vs. Model group. YSA, Yi Shen An. PRE, prednisone acetate positive control.

Changes in proteinuria were investigated on days 14, 28, and 35 (Figure 3). All groups that received treatment showed reduced levels of 24 h urinary protein (to various degrees) compared with the corresponding levels observed in the model group. Of note, the greatest reduction in the levels of urinary protein was observed on days 28 and 35 in all groups (*p* < 0.01).

### Biochemicals changes in the blood

Compared with the control group, the levels of TP and ALB in the C-BSA-induced group were significantly lower (*p* < 0.05) while the levels of TG and TC were significantly higher (*p* < 0.01) (Table 2). Of note, the levels of CRE and BUN were not significantly different when compared between the C-BSA-induced group and the control group (*p* > 0.05). However, the levels of TP and ALB had increased significantly in the YSA groups when compared to the model group, whereas TG and TC levels had decreased to almost normal levels (*p* < 0.05/0.01). The positive control group (PRE) showed the same effect as YSA except that the TG level showed a non-significant tendency to decrease (*p* > 0.05).

**Table 2.** The effects of YSA on serum biochemical parameters.

Group	TC (mmol/L)	TG (mmol/L)	BUN (mmol/L)	Cre (µmol/L)	TP (g/L)	ALB (g/L)
Control	1.40 ± 0.06	0.53 ± 0.04	6.51 ± 0.47	36.37 ± 4.48	76.32 ± 2.04	37.60 ± 0.71
Model	3.31 ± 0.54^##^	1.58 ± 0.39^#^	5.86 ± 0.24	29.58 ± 1.31	66.26 ± 2.32^##^	30.51 ± 1.11^##^
PRE	1.81 ± 0.25*	1.01 ± 0.24	5.91 ± 0.27	29.71 ± 1.23	76.25 ± 1.99**	35.87 ± 0.67**
YSA-0.25	1.69 ± 0.06*	0.65 ± 0.07*	5.63 ± 0.30	30.38 ± 1.64	75.17 ± 2.01**	35.07 ± 0.64**
YSA-0.5	1.78 ± 0.13*	0.55 ± 0.06*	6.08 ± 0.24	29.43 ± 2.02	76.08 ± 1.43**	34.48 ± 0.50**
YSA-1	1.65 ± 0.12*	0.58 ± 0.04*	6.90 ± 0.81	41.61 ± 9.23	74.71 ± 1.08**	35.64 ± 0.45**
YSA-2	1.78 ± 0.07*	0.67 ± 0.07*	7.37 ± 0.75	33.72 ± 3.08	75.25 ± 1.44**	35.69 ± 0.46**

Data are shown as means ± standard error of the mean. Statistical differences are represented as ^##^*p* < 0.01 vs. Control, ^*/**^p < 0.05/0.01 vs. Model group. YSA, Yi Shen An; PRE, prednisone acetate positive control; TC, total cholesterol; TG, triglyceride; BUN, blood urea nitrogen; CRE, creatinine; TP, total protein; ALB, albumin. YSA-0.25, 0.5, 1, and 2 represent rats that were orally administrated with YSA at doses of 0.25, 0.5, 1, and 2 g/kg, respectively.

### Effects on the levels of CIC, IgG, IgM and IgA

**Figure 4.**
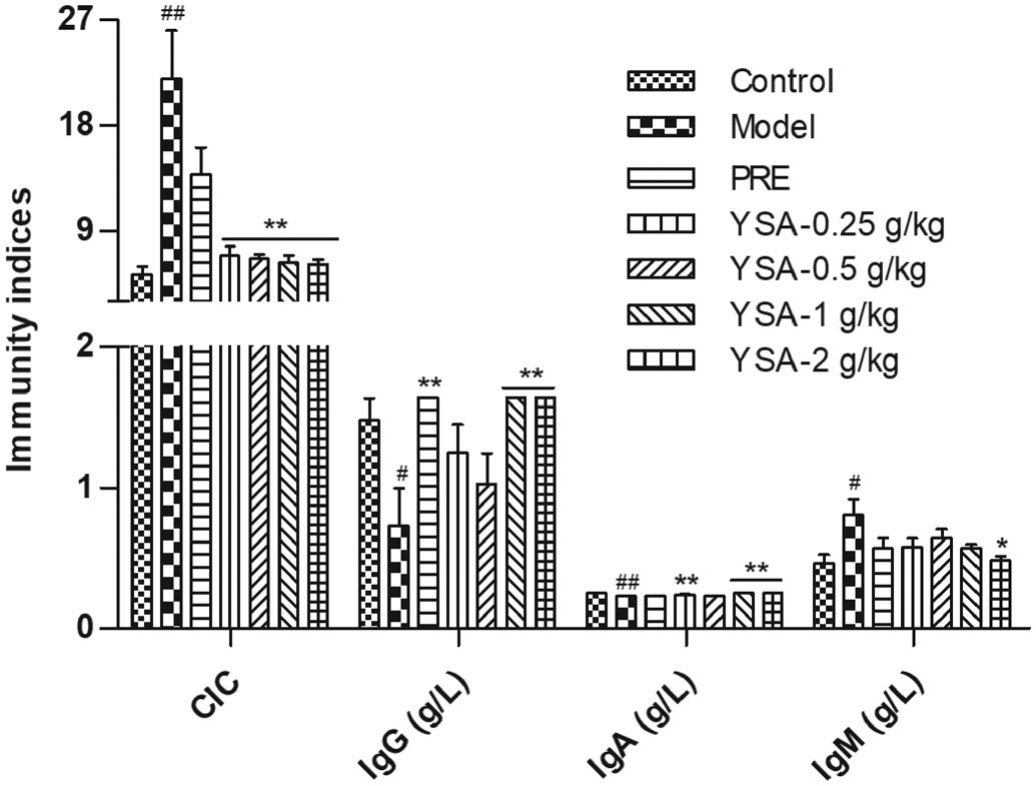
Bar graphs representing the levels of CIC, IgG, IgA, and IgM. Statistical differences are represented as ^#/##^*p* < 0.05/0.01 vs. Control, ^*/**^p < 0.05/0.01 vs. Model group. CIC, circulating immune complex; IgG, immunoglobulin G; IgA, immunoglobulin A; IgM, immunoglobulin M; YSA, Yi Shen An; PRE, prednisone acetate positive control.

In the model group, the serum levels of CIC and IgM were significantly higher than those in the control group (*p* < 0.05/0.01, Figure 4). In contrast, the levels of IgA and IgG were significantly lower than those in the control group (*p* < 0.05/0.01). As expected, the levels of CIC were reduced following the oral administration of YSA for 35 days (*p* < 0.01). Moreover, the levels of IgM tended to gradually decrease in the YSA-treated groups. Of note, the YSA 2 g/kg group showed the most pronounced change compared with the model group (*p* < 0.05). Similarly, the levels of IgA and IgG were markedly increased to almost normal levels in both 1 and 2 g/kg groups (*p* < 0.01). However, in the PRE group, only the levels of IgG showed a significant increase (*p* < 0.01).

### The effects of YSA on blood coagulation

In the model group, the levels of FIB and TT were markedly higher than in the control group (*p* < 0.05/0.01, Table 3). In contrast, there were no apparent changes in the levels of APTT and PT. However, all animals treated with YSA showed lower levels of FIB contents than the model group (*p* < 0.05/0.01). Moreover, the levels of TT and PT tended to decrease gradually in the YSA-treated groups. Of note, the 0.5 and 1 g/kg groups showed the most pronounced changes in TT when compared with the model group. In addition, the 0.25 and 1 g/kg groups showed the most pronounced changes in terms of PT levels when compared with the model group (*p* < 0.05/0.01). In the PRE group, we identified significant changes in the levels of TT and FIB (*p* < 0.05/0.01).

**Table 3.** The effects of YSA on coagulation indexes.

Group	APTT (s)	TT (s)	PT (s)	FIB	
				Duration (s)	Content (g/L)
Control	22.98 ± 1.81	23.55 ± 0.85	16.04 ± 0.15	19.03 ± 1.24	1.86 ± 0.16
Model	21.18 ± 1.50	31.12 ± 1.47^##^	16.89 ± 0.40	10.37 ± 0.65^##^	3.86 ± 0.27^##^
PRE	19.65 ± 0.71	27.95 ± 0.74*	16.92 ± 0.30	13.65 ± 0.56**	2.71 ± 0.16**
YSA-0.25	20.59 ± 1.28	28.97 ± 3.21	13.80 ± 1.34*	15.73 ± 1.98*	2.56 ± 0.26**
YSA-0.5	21.83 ± 0.84	24.65 ± 0.93**	15.55 ± 0.66	15.85 ± 1.14**	2.37 ± 0.19**
YSA-1	25.23 ± 1.15	26.18 ± 1.24*	15.59 ± 0.21*	16.16 ± 0.91**	2.24 ± 0.14**
YSA-2	25.45 ± 2.60	27.97 ± 0.64	16.29 ± 0.31	20.28 ± 1.27*	2.08 ± 0.25**

Data are shown as means ± standard error of the mean. Statistical differences are represented as ^##^*p* < 0.01 vs. Control, ^*/**^p < 0.05/0.01 vs. Model group. YSA, Yi Shen An; PRE, prednisone acetate positive control; APTT, activated partial thromboplastin; TT, thrombin time; PT, prothrombin time; FIB, fibrinogen. YSA-0.25, 0.5, 1, and 2 represents rats that were orally administrated with YSA at doses of 0.25, 0.5, 1, and 2 g/kg, respectively.

### The effects of YSA on oxidative stress

**Figure 5.**
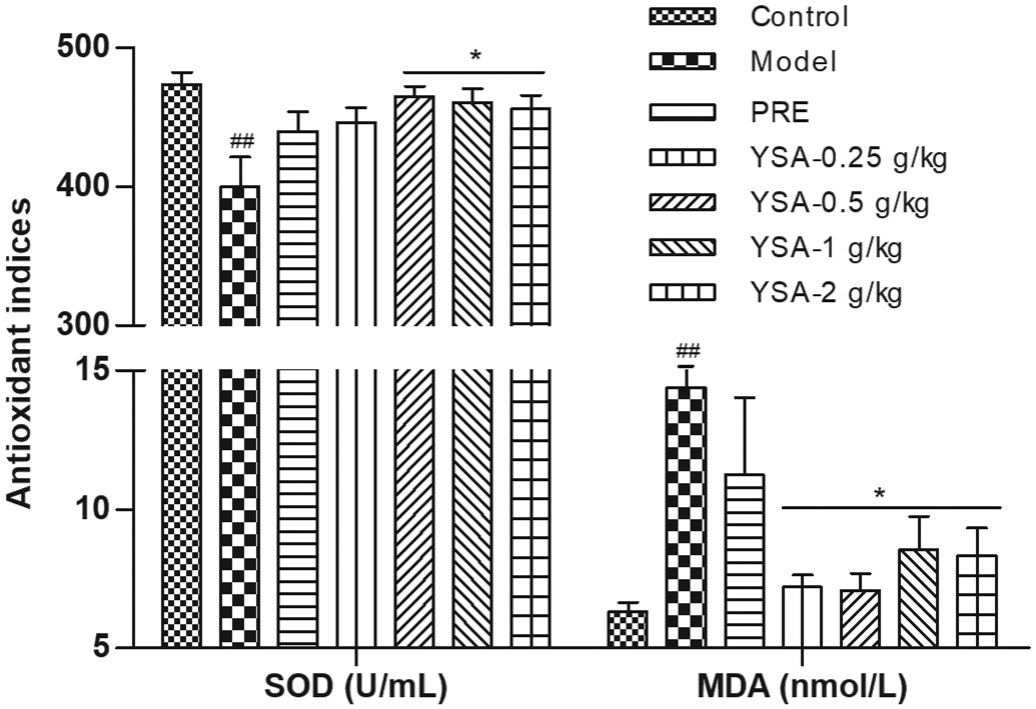
Bar graphs representing the levels of SOD and MDA. Statistical differences are represented as ^##^*p* < 0.01 vs. Control, *p < 0.05 vs. Model group. SOD, superoxide dismutase; MDA, malondialdehyde; YSA, Yi Shen An; PRE, prednisone acetate positive control.

As shown in Figure 5, the SOD activity in the serum of animals belonging to the model group was significantly reduced whereas the levels of MDA were significantly higher than those reported in the control group (*p* < 0.01). In contrast, after the administration of YSA, the SOD activity increased significantly whereas the levels of MDA decreased significantly when compared to the model group (*p* < 0.01). In the PRE group, the SOD activity, and levels of MDA, showed a tendency towards elevation and reduction, respectively, when compared with those observed in the model group (*p* > 0.05).

### Pathological observation of renal tissue

**Figure 6.**
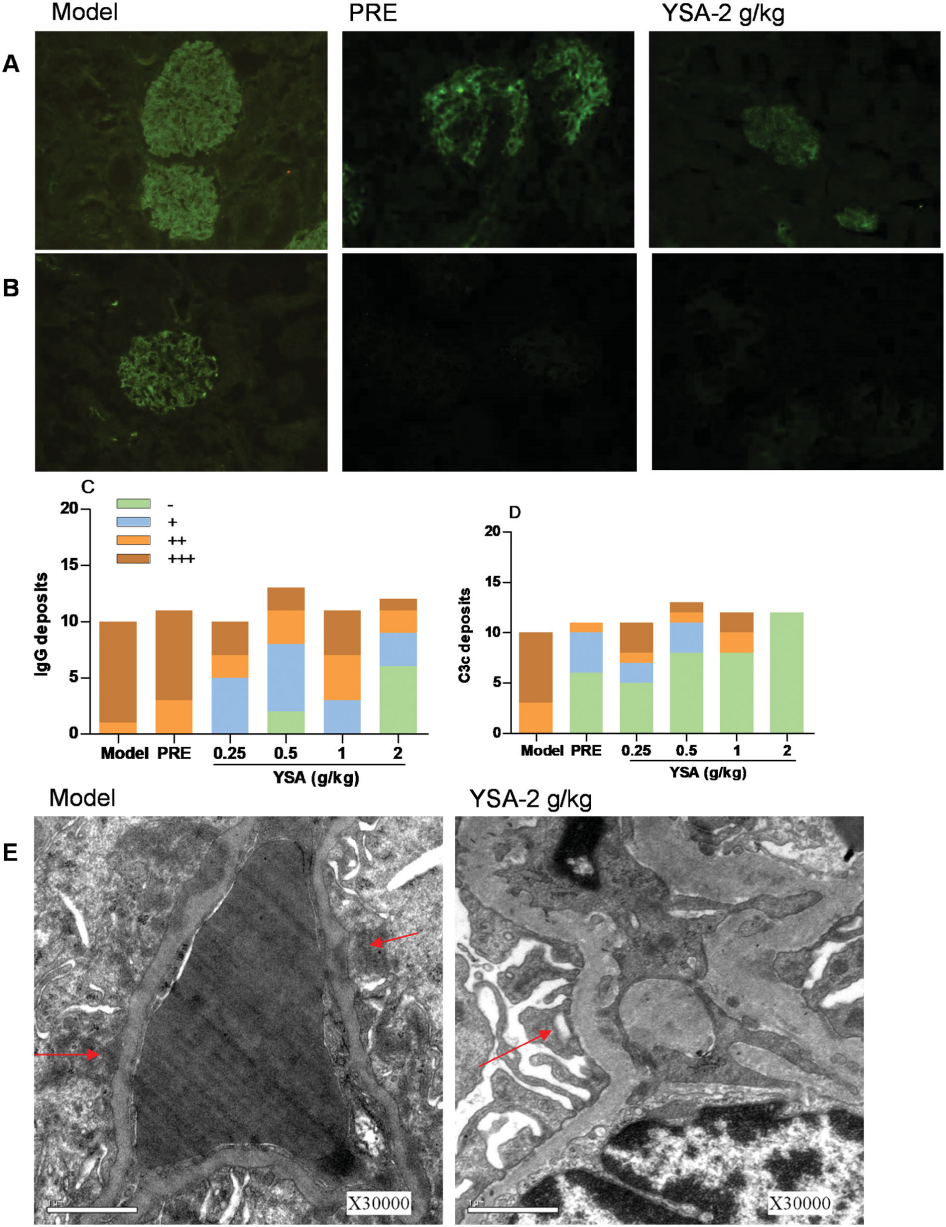
Renal immunofluorescence staining and scanning electron microscope analysis of CBSA-induced MGN rats. YSA, Yi Shen An; PRE, prednisone acetate positive control. Statistical differences are represented as ^##^*p* < 0.01 vs. Control, *p < 0.05 vs. Model group. (A) Immunohistochemical staining of immunoglobulin G (IgG, original magnification ×20); (B) Immunohistochemical staining of complement 3c (C3c, original magnification ×20); (C, D) Quantitative analysis of IgG (C) and C3c deposits (D); (E) Transmission electron micrographs of renal tissue. (Original magnification ×30,000). ‘→’ represents the presence of glomerular podocyte fusion, the proliferation of mesangial cells and matrix, and electron-dense deposits in the GBM and epithelia.

**Figure 7.**
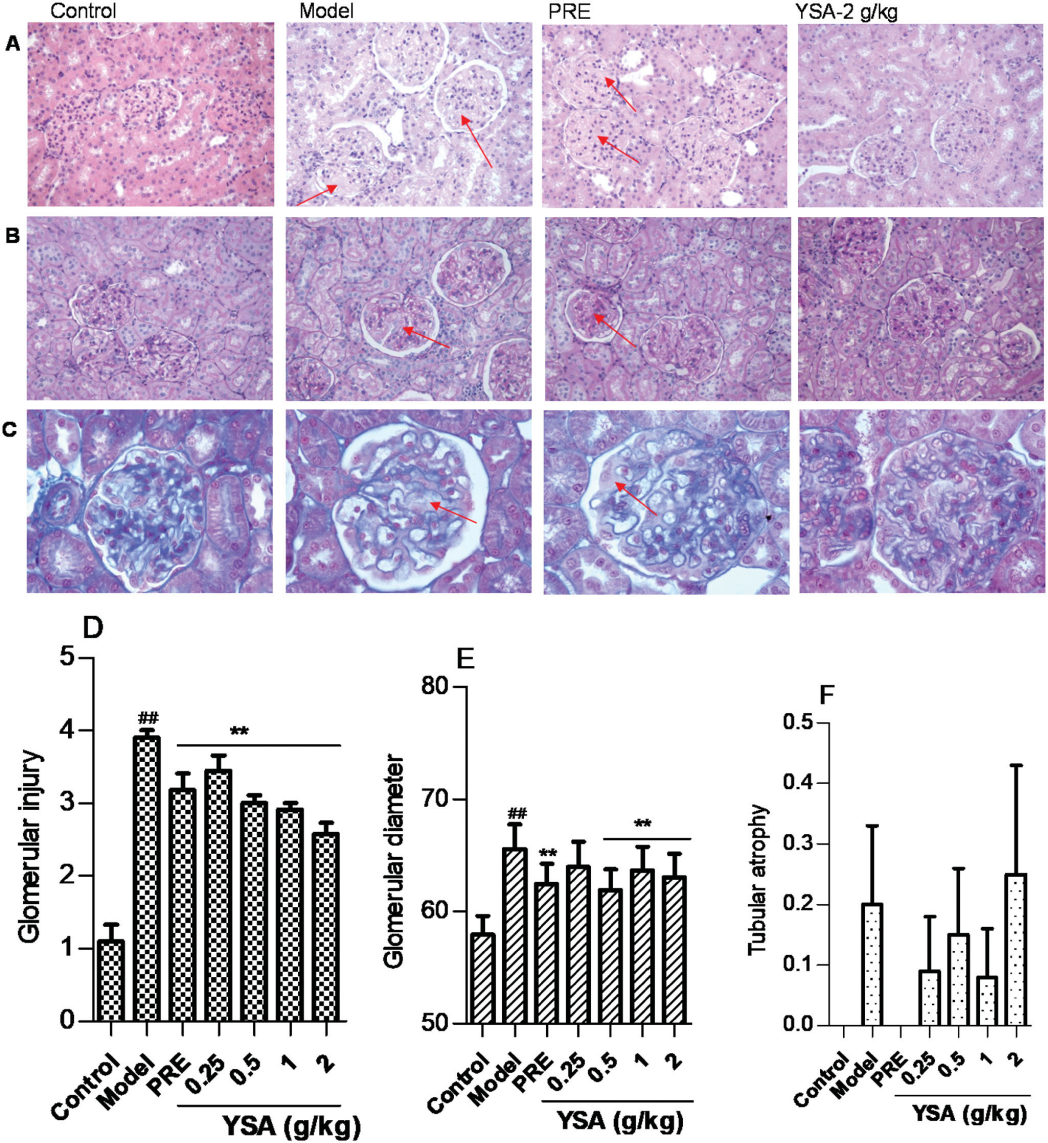
The effects of YSA on histopathological changes in the kidney tissues of MGN rats. At the end of the study, rat kidneys were harvested and fixed using 4% neutralized formalin. Paraffin sections were then prepared and stained with haematoxylin & eosin, periodic acid-Schiff, and Masson’s trichrome staining. A double-blinded method was used in the pathological analysis, the results were qualitatively identical, and representative results are shown. YSA, Yi Shen An; PRE, prednisone acetate positive control. (A) Representative haematoxylin-eosin staining of kidney samples (original magnification ×40). ‘→’ represents hyperplasia of mesangial cells and the matrix, as well as the luminal stenosis of renal capsule and capillaries. (B) Representative periodic acid-Schiff staining of kidney sections (original magnification ×40). ‘→’ represents glomerular mesangial cell proliferation and basement membrane thickness. (C) Representative Masson’s trichrome staining of kidney sections (original magnification ×100). ‘→’ represents the deposition of polytrophin in the mesangial area. (D–F) The scores for glomerular injury (D), glomerular diameter (E) and tubular atrophy (F). Statistical differences are represented as ^##^*p* < 0.01 vs. Control, **p < 0.01 vs. Model group.

Immunofluorescence staining showed that IgG and C3c were strongly expressed in the mesangium and GBM in the model group (Figure 6(A–D)). Interestingly, both IgG and C3c were expressed at low levels in the YSA groups (to varying degrees). Moreover, electron microscopy revealed the presence of glomerular podocyte fusion, the proliferation of mesangial cells and matrix, and electron-dense deposits in both the GBM and epithelia (Figure 6(E)). However, treatment with YSA rescued these pathological changes, to varying extents. Compared with the model group, the 2 g/kg group showed the most prominent changes. However, the PRE group demonstrated a significant scavenging effect on C3c, without influencing the levels of IgG. H&E staining revealed that the glomeruli of animals treated with C-BSA were associated with diffuse distribution and increased volume. In addition, the animals treated with C-BSA exhibited hyperplasia of the mesangial cells and the matrix, as well as luminal stenosis in the renal capsule and capillaries (Figure 7(A)). PAS staining demonstrated glomerular mesangial cell proliferation and an increase in the thickness of the basement membrane (Figure 7(B)). Furthermore, Masson staining demonstrated the deposition of polytrophin in the mesangial area (Figure 7(C)). Collectively, these anomalies indicated that the model closely resembled human mesangial proliferative glomerulonephritis. The scores for glomerular injury, tubular atrophy, and glomerular diameter are shown in Figure 7(D–F). Histological changes in the model group were more serious than those in the control group (*p* < 0.01). However, these changes were modest in the YSA-treated group; the most pronounced improvements related to glomeruli injury and glomerular diameter (*p* < 0.01).

## Discussion

Membranous glomerulonephritis (MGN) is a glomerular disease with distinct features in which the gradual accumulation of immunoglobulin and complement occurs in a discontinuous granular pattern in the subepithelial space, thus resulting in a marked increase in capillary permeability with proteinuria and nephrotic syndrome (Salant et al. 1980; Lai et al. 2015). Cationic bovine serum albumin (C-BSA)-induced glomerulonephritis, an animal model of immune complex glomerular injury, was previously developed to investigate the immunological mechanisms of human glomerulonephritis. This model has been used to investigate a variety of experimental animals and has been shown to exhibit features that are characteristic of human glomerulonephritis (Wu et al. 2009; Wang et al. 2016). Pathogenesis occurs due to the binding of positively charged C-BSA to negatively charged GBM, thus resulting in the formation of immune complexes (e.g., IgG, IgA, and IgM) in the glomeruli (Doi et al. 1984; Huang et al. 2013; Motiram et al. 2016). These complexes may trigger the complement system and result in glomerular injury, eventually leading to the induction of glomerulonephritis and impaired renal function (Couser 1985; Paccaud and Schifferli 1989). Approximately half of the patients with MGN exhibit positive staining for C3, predominantly C3c, a short-lived breakdown product of C3, thus indicating the ongoing formation of immune deposits and active disease (Nangaku et al. 2005).

Histopathological changes and evaluation scores for the treatment of glomerulonephritis directly reflect the severity of animal models and the effects of drug interventions. In the present study, we found that the surface of the kidney was pale and the kidney index was significantly increased. Moreover, immunofluorescence examination revealed high expression levels of IgG and C3c in the model group. However, the degree of renal tissue damage was alleviated in the YSA group; our data suggest that the mechanisms underlying these effects may be related to the clearance of IgG and C3c. We also observed reduced serum levels of IgG and increased levels of IgM and CIC. These results indicated that YSA may regulate the observed abnormalities in these key indices.

Podocytes are highly specialized epithelial cells that cover the outer layer of the GBM. Previous studies have demonstrated a close connection between podocytes and adjacent components within the glomerular filtration barrier, especially endothelial cells (Cheng and Harris 2010). The damage or loss of podocytes may allow protein molecules to pass through the surface of the GBM, thus resulting in proteinuria (Reiser and Sever 2013; Ma et al. 2015). Therefore, a reduced number of podocytes is a key determinant underlying the progression of nephrotic syndrome to renal failure (Wu et al. 2014). In the present study, we used electron microscopy to observe the fusion of foot processes and the proliferation of mesangial cells and matrix in the model group. However, these changes were rescued following the administration of YSA for 35 days. Heavy proteinuria is a marker and a key risk factor for various nephrotic diseases (Wu et al. 2014); consequently, the reduction of proteinuria is a key therapeutic goal in clinical practice (Orth and Ritz 1998). Following the injection of C-BSA, we observed that protein excretion increased progressively and was significantly suppressed after the administration of YSA. Our results showed that YSA significantly inhibited the fusion of podocytes, and proteinuria, thus improving renal function.

Kidney function is directly related to the level of protein excretion in the urine (Jiang et al. 2015). Serum ALB, the most highly expressed protein in plasma, serves as a carrier protein for steroids, fatty acids, and thyroid hormones, and plays a key role in maintaining colloid osmotic pressure (Wang et al. 2016). ALB can be excreted into the urine when the pore size of the GBM is increased, thus allowing greater filtration. This also increases the transport of small molecular weight proteins in the plasma and results in a reduction of charge in the barrier and alterations in the re-uptake ability of the renal tubules (Pavenstädt et al. 2003). These changes may also cause hypoalbuminemia (Stahl and Hoxha 2016). In addition, a reduction in the plasma protein levels in the body can stimulate the synthesis of plasma protein in the liver. Increased levels of lipoprotein cause an increase in the levels of low-density lipoprotein (LDL) and the synthesis of TC in the liver. Moreover, reduced activity of the LDL receptor causes excessive binding of LDL to excess lipoprotein, thus leading to hyperlipemia (Oda and Keane 1997). In the present study, the administration of YSA in rats with C-BSA-induced MGN reduced the total amount of urinary protein and increased the levels of TP and ALB. Furthermore, the administration of YSA also reduced hypercholesteremia and hypertriglyceridaemia, and improved renal function.

Thromboembolism is one of the most serious complications of MGN (Orth and Ritz 1998; Kerlin et al. 2012). Coagulation disorders, and changes in hemorheology, play an important role in the occurrence and development of thromboembolism. The loss of a large amount of protein can lead to further reductions in the levels of anticoagulants and plasminogen (Llach 1985). Reduced plasma colloid osmotic pressure and renal blood flow, increased plasma viscosity, and reduced blood concentration, can all lead to the formation of microthrombi in the glomerular capillaries (Mahmoodi et al. 2008). A high state of coagulation in the blood can also promote glomerular damage, eventually leading to chronic renal failure (Barbour et al. 2012). The levels of PT reflect the levels of coagulation factors in the extrinsic pathway, whereas levels of APTT reflect abnormalities in the intrinsic pathway. The prolongation of APTT is due to deficiencies in factors within the intrinsic pathway or a common pathway (Ali et al. 2016). We found that the administration of YSA promoted blood circulation, as demonstrated by the reduction in serum FIB and the shortening of the TT. In addition, YSA exerted notable effects on APTT and PT.

The main sources of oxygen-free radicals in kidney tissues are kidney intrinsic cells and infiltrating cells. The dynamic balance between the antioxidant capacity of renal tissue and the oxidative capacity of free radicals is known to be impaired following damage to the kidney tissue (Kashem et al. 1996). Excessive levels of oxygen-free radicals may cause direct damage to the endothelium of the glomeruli and mesangial cells, thus resulting in the shedding of endothelial cells, thereby attracting the infiltration of neutrophils and mononuclear giant cells around and inside blood vessels. This can also cause the release of cytokines and inflammatory mediators, further aggravating the damage incurred by the glomerular cells (Song et al. 2016; Daenen et al. 2019). Antioxidant enzymes, such as SOD and MDA, are markers of oxidation-induced tissue damage (Motiram et al. 2016). SOD is an enzyme that converts superoxide to hydrogen peroxide and plays a critical role in reducing oxidative stress and inflammatory responses (Carillon et al. 2013). MDA is a product formed by lipid peroxidation and represents a biomarker of oxidative stress (Stancliffe et al. 2011). Our results demonstrated that the administration of YSA significantly inhibited C-BSA-induced MGN by inhibiting MDA activity and enhancing SOD activity. However, our present research focussed on the protective effect of YSA on MGN for the application of clinical trials. The potential mechanisms involved in the effects of a new drug play a key role in their pharmacological development. Further biological studies are now needed to identify the target(s) and therapeutic mechanism of YSA, both *in vivo* and *in vitro*. The findings of the present study are highly valuable in that they can promote the development and application of YSA. It is worth noting that the positive control used in the present study, prednisone, an immunosuppressant of glucocorticoid, also showed a significant protective effect against MGN. However, prednisone is associated with side effects, particularly weight loss; these effects were not observed with YSA treatment. Furthermore, we found that YSA was superior to prednisone in terms of the reduction of triglycerides, improving blood circulation and antioxidant activity, and the clearance of IgG. We speculate that YSA represents a better treatment option for MGN due to the synergistic effects of multiple components, multiple targets, and multiple pathways.

Previous studies reported that glomerulonephropathy is the most common factor responsible for the development of the nephritic syndrome. There are few effective drugs available in clinical practice for the treatment of this condition (Wu et al. 2016). Traditional Chinese medicine (TCM) is one of China’s national essences and has been used for thousands of years to treat incurable diseases. The prescription of TCM agents, in strict accordance with the recommendations made by the pharmacopoeia (i.e., paying close attention to its origin, dosage, method of preparation, and the duration of intake), may result in reduced toxicity and fewer adverse effects (Liu et al. 2009). The formulation of YSA capsules includes 10 different herbs to exert a curative effect and reduce toxicity. In our preclinical safety evaluation of YSA, we performed chronic toxicity tests in rats for 26 weeks. We observed no mortality in YSA-treated rats, no adverse effects, and no dose-dependent changes in terms of biochemistry and histopathology at doses of 1, 2 or 5 g/kg except for an increase in urinary output in the 5 g/kg group. The maximum dosage of YSA was 32 g/kg in acute toxicity tests; this did not result in obvious toxicity or death. Furthermore, seven chemical components were identified as standards for quality control, including *P. notoginseng* saponins R1, ginseng saponin Rg1, ginsenoside Rb1, tanshinone IIA, emodin, aloe-emodin and chlorogenic acid. Of these, *P. notoginseng* was identified as the principal-agent and has been shown to exert renoprotective effects against tubule-interstitial fibrosis induced by adenine (Zhao et al. 2008). Moreover, notoginsenoside R1, ginsenoside Rg1 and Rb1, the major active component of saponins, have been shown to exert renoprotective effects against diabetic nephropathy by inhibiting the apoptosis and renal fibrosis caused by oxidative stress (Zhang et al. 2019). These components also inhibit the transition of tubular epithelial cells into myofibroblasts (Xie et al. 2009), along with LPS-induced inflammation and apoptosis in HK-2 cells (Ni et al. 2017). In addition, *S. miltiorrhiza*, one of the most famous TCM herbal agents, was found to maintain tubular function and structure (Yin et al. 2014) and reverse renal injury induced by myocardial infarction (Lu et al. 2015). Tanshinone IIA, a diterpenoid quinone, is the most crucial fat-soluble ingredient of *S. miltiorrhiza*; previous research showed that this component protected rats from uric acid-induced kidney damage *via* NF-κB inhibition (Wu et al. 2012). Emodin, the most abundant anthraquinone of *R. officinale* was shown to reduce urinary protein levels in a mouse model of lupus nephritis (Yuan et al. 2020) and also inhibited the proliferation and promoted the apoptosis, of human kidney fibroblasts (Liu et al. 2000). Aloe-emodin was also shown to ameliorate renal fibrosis in mice induced by unilateral ureteral obstruction by inhibiting the PI3K/Akt/mTOR signalling pathway (Dou et al. 2019). Chlorogenic acid is the ester of caffeic acid and-quinic acid and the main component of *L. confuse*; this has been shown to prevent diabetic nephropathy by inhibiting oxidative stress and inflammation (Bao et al. 2018). Therefore, the clinical improvements observed following the administration of YSA may be attributed to the synergistic effect of multiple components. Of these, the saponin of *P. notoginseng* appears to play a leading role in the protective effects observed. Further studies are now warranted to identify the major active constituents in YSA that are responsible for the observed beneficial effects in the MGN.

## Conclusions

This study is the first to show that YSA can alleviate renal structural injury, proteinuria, and serum lipid levels in rats injected with C-BSA. YSA also improved blood hypercoagulability and the imbalance of oxidation-antioxidation. The therapeutic mechanism underlying these actions may be related to the inhibition of CIC formation, and the removal of IgG and C3c deposits from the GBM. These findings provide a novel candidate drug for the treatment of MGN.

## Supplementary Material

Supplemental Material
