# Supplementary material for: Yi Shen An, a Chinese traditional prescription, ameliorates membranous glomerulonephritis induced by cationic bovine serum albumin in rats
Source: Pharm Biol. 2022 Jan 8;60(1):163–74. doi: 10.1080/13880209.2021.2021947 (PMC8745358; doi:10.1080/13880209.2021.2021947)
Supplement: Supplemental Material [file IPHB_A_2021947_SM4844.docx]

**Supporting Information**

YSA is composed with ten herbs. First, *S.* *miltiorrhiza* (240 g) was extracted twice using six volumes of 90% ethanol under reflux conditions (1.5 h per replicate). The solvent was then evaporated under vacuum to obtain an ethanol extract (36 g). Next, *R. officinale* (180 g) was extracted twice using six volumes of 60% alcohol for 1.5 h per replicate). The solvent was then evaporated under vacuum to obtain an ethanol extract (65 g). The seven remaining medicinal materials (i.e., *L. confusa* 120 g; *C.* *tinctorius* 100 g; *F.* *suspensa* 100 g; *S.* *barbata* 120 g; *W. extensa* 150 g; *P. lingua* 100 g, and *G.* *uralensis* 80 g) were soaked in 12 volumes of distilled water for decoction (1 h × two replicates) followed by extraction with 95% ethanol. Then, the solvent was evaporated under vacuum to obtain an ethanol extract (18 g). Finally, 160 g of *P.* *notoginseng* powder was mixed with the three dry extracts and ground into powder. Each gram of YSA extract contained 0.27 g of crude drug.


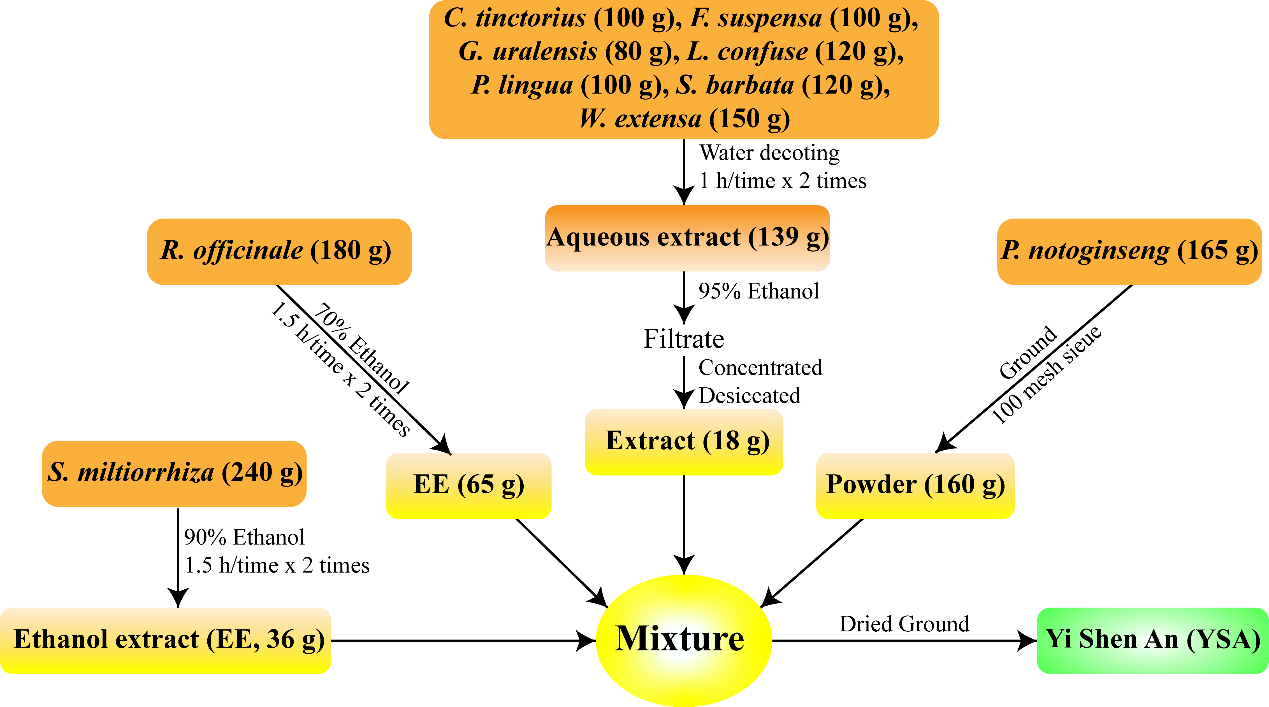


**Fig. S1** The preparation processes of Yi Shen An (YSA)
